# Deceased Donor Kidneys Utilization and Discard Rates During COVID-19 Pandemic in the United States

**DOI:** 10.1016/j.ekir.2021.06.002

**Published:** 2021-06-19

**Authors:** Miah T. Li, Kristen L. King, S. Ali Husain, Jesse D. Schold, Sumit Mohan

**Affiliations:** 1 Division of Nephrology, Department of Medicine, Vagelos College of Physicians and Surgeons, Columbia University, New York, New York, USA; 2 Columbia University Renal Epidemiology Group, New York, New York, USA; 3 Department of Epidemiology, Mailman School of Public Health, Columbia University, New York, New York, USA; 4 Department of Quantitative Health Sciences, Cleveland Clinic, Cleveland, Ohio, USA; 5 Center for Populations Health Research, Lerner Research Institute, Cleveland Clinic, Cleveland, Ohio

**Keywords:** COVID-19, deceased donor kidney, discard, utilization

The COVID-19 pandemic created significant disruptions to kidney transplantation in the United States. Particularly in the initial surge, there was a dramatic decrease in deceased donor transplants, a near complete cessation in living donor kidney transplants, and considerable mortality among kidney transplant candidates.^1^, ^2^, ^3^ Although some of the significant operational challenges for organ procurement organizations and transplant centers have been described,^4^ remarkably more deceased donor kidney transplants occurred in 2020 than in any previous year. However, the impact of the pandemic on organ procurement and utilization are unclear. In this study, we evaluated the impact of the COVID-19 pandemic on deceased donor kidney utilization and discard rates in the United States in 2020 using national registry data as of January 1, 2021 (Supplementary Methods).

## Results

### Procurements and Discards

The overall number of deceased donors dropped dramatically during the initial COVID-19 surge (March 18–May 12, 2020) from a mean (SD) of 252 (±15) donors weekly presurge to a low of 156 donors the week beginning March 25, 2020 (Supplementary Figure S1). From an already reduced number of deceased donors, 168 kidneys (6%) that were consented for transplantation were not even procured during the initial surge (Table 1). The weekly kidney discard rate among procured kidneys was highest during the initial surge, dropped after the surge, and rose mildly in the summer and again at the end of the year, coinciding with different waves of COVID-19 infections in 2020 instead of following the relatively flat trend as observed in 2019 (Supplementary Figures S1 and S2). During the initial surge, up to 29% of the kidneys recovered weekly for transplantation were discarded. The annual discard rate in 2020 was 21% (5051 kidneys), with regional variation (Supplementary Figure S3). Overall organ disposition varied significantly by Organ Procurement and Transplant Network (OPTN) region both during the initial surge and for all of 2020 (both *P* < 0.001; Table 1). During the initial surge, the proportion of consented donor kidneys that were not transplanted ranged from a high of 31% discarded and 15% not procured in region 9 to a low of 18% discarded and 2% not procured in region 10.

**Table 1:** Deceased donor characteristics by organ dispositions during the initial surge versus all of 2020

Donor characteristics	All of 2020			Initial surge: Weeks 12–19		
	Transplanted, *n* (%) (*n* = 18,686; 74.8%)	Discarded, *n* (%) (*n* = 5051; 20.2%)	Not procured, *n* (%) (*n* = 1250; 5.0%)	Transplanted, *n* (%) (*n* = 2,165; 72.5%)	Discarded, *n* (%) (*n* = 654; 21.9%)	Not procured, *n* (%) (*n* = 168; 5.6%)
Age, median (IQR)	38 (27–50)	56 (45–62)	54 (43–64)	36 (27–48)	54 (40–60)	51 (36–63)
Gender						
Female	6813 (72)	2215 (23)	495 (5)	794 (69)	288 (25)	72 (6)
Male	11,873 (77)	2836 (18)	755 (5)	1371 (75)	366 (20)	96 (5)
Race/ethnicity						
White	12,323 (75)	3487 (21)	599 (4)	1448 (74)	437 (22)	79 (4)
Black	2709 (69)	806 (21)	394 (10)	307 (65)	111 (24)	52 (11)
Hispanic	2880 (79)	561 (15)	214 (6)	309 (74)	84 (20)	26 (6)
Other	774 (76)	197 (19)	43 (4)	101 (75)	22 (16)	11 (8)
Body mass index, median (IQR)	27.2 (23.3–32.0)	28.7 (24.4–34.3)	28.2 (24.7–33.9)	27.0 (23.2–31.8)	28.0 (23.8–33.4)	27.4 (23.4–31.8)
Donation after circulatory death	4792 (74)	1562 (24)	82 (1)	454 (73)	165 (26)	5 (1)
HCV NAT^a^	1208 (74)	373 (23)	55 (3)	157 (73)	50 (23)	9 (4)
CMV^a^	11,407 (74)	3213 (21)	849 (5)	1283 (71)	396 (22)	120 (7)
COVID-19 NAT or other test positive	13 (46)	15 (54)	0 (0)	0 (0)	0 (0)	0 (0)
COVID-19 antibody test positive	18 (75)	6 (25)	0 (0)	0 (0)	0 (0)	0 (0)
Terminal creatinine, median (IQR)	0.9 (0.7–1.4)	1.5 (0.9–2.8)	4.1 (1.7–6.8)	0.9 (0.7–1.4)	1.5 (0.9–2.9)	3.9 (1.7–7.2)
Proteinuria						
Yes	9815 (71)	3187 (23)	905 (7)	1126 (69)	388 (24)	113 (7)
No	8771 (81)	1816 (17)	243 (2)	1028 (77)	259 (19)	43 (3)
Unknown or missing	100 (40)	48 (19)	102 (40)	11 (37)	7 (23)	12 (40)
History of hypertension	5016 (56)	3105 (34)	915 (10)	557 (53)	368 (35)	120 (11)
History of diabetes	1485 (45)	1286 (39)	504 (15)	162 (47)	125 (36)	56 (16)
Cigarette use (past or present)	3622 (65)	1651 (30)	298 (5)	429 (66)	173 (27)	45 (7)
History of i.v. drug use	2708 (82)	497 (15)	88 (3)	361 (80)	71 (16)	18 (4)
Public Health Service–Increased Risk (PHS-IR)	5309 (77)	1089 (16)	511 (7)	665 (75)	147 (17)	72 (8)
Kidney Donor Risk Index (KDRI)–Rao, median (IQR)	1.2 (1.0–1.5)	1.8 (1.5–2.2)	2.0 (1.6–2.5)	1.1 (0.9–1.4)	1.7 (1.4–2.1)	1.9 (1.5–2.4)
Kidney Donor Profile Index (KDPI), median (IQR)	42 (21–65)	83 (65–93)	89 (71–97)	39 (19–62)	77 (59–91)	86 (68–96)
Biopsy performed						
Yes	9352 (68)	4450 (32)	3 (0)	944 (64)	537 (36)	0 (0)
No	9332 (86)	600 (6)	903 (8)	1219 (84)	117 (8)	110 (8)
Missing	2 (1)	1 (0)	344 (99)	2 (3)	0 (0)	58 (97)
OPTN region						
1	563 (78)	140 (19)	15 (2)	66 (77)	20 (23)	0 (0)
2	2053 (72)	656 (23)	150 (5)	246 (69)	87 (24)	23 (6)
3	2857 (74)	729 (19)	281 (7)	316 (71)	95 (21)	31 (7)
4	1879 (75)	514 (21)	107 (4)	241 (74)	71 (22)	14 (4)
5	2952 (76)	801 (20)	155 (4)	362 (68)	142 (27)	26 (5)
6	849 (81)	170 (16)	25 (2)	99 (79)	25 (20)	2 (2)
7	1419 (77)	309 (17)	118 (6)	138 (73)	27 (14)	25 (13)
8	1471 (74)	416 (21)	103 (5)	159 (76)	36 (17)	13 (6)
9	634 (69)	213 (23)	66 (7)	44 (54)	25 (31)	12 (15)
10	1751 (76)	482 (21)	85 (4)	219 (79)	51 (18)	6 (2)
11	2258 (75)	621 (21)	141 (5)	275 (75)	75 (20)	16 (4)

CMV, cytomegalovirus; HCV, hepatitis C virus; IQR, interquartile range; NAT, nucleic acid amplification test; OPTN, organ procurement and transplantation network.

Unless otherwise noted, values are *n* (row %). Missing values excluded were as follows: body mass index (50), hypertension (348), diabetes (350), cigarette use (676), i.v. drug use (474), and KDRI/KDPI (2).

a HCV NAT and CMV are counted if the test results were positive, indeterminate, pending, or not done.

More kidneys were discarded from donors who were older and female, and donors with higher terminal creatinine and a history of diabetes, hypertension, or smoking history, but kidneys from donors with a history of drug use and Public Health Service–Increased Risk status were more frequently transplanted (Table 1). More than half (52%) of the discards in 2020 resulted from a failure to identify a recipient, with a high of 60% at the peak of the initial surge in April compared to an average of 47% between January and February 2020 (Supplementary Figure S4). Approximately 1 in 5 discards were attributed to poor biopsy findings, although this was less common during the height of the initial surge, accounting for only 12% of discards in April (Supplementary Figure S4).

### Transplant Center Utilization

**Figure 1:**
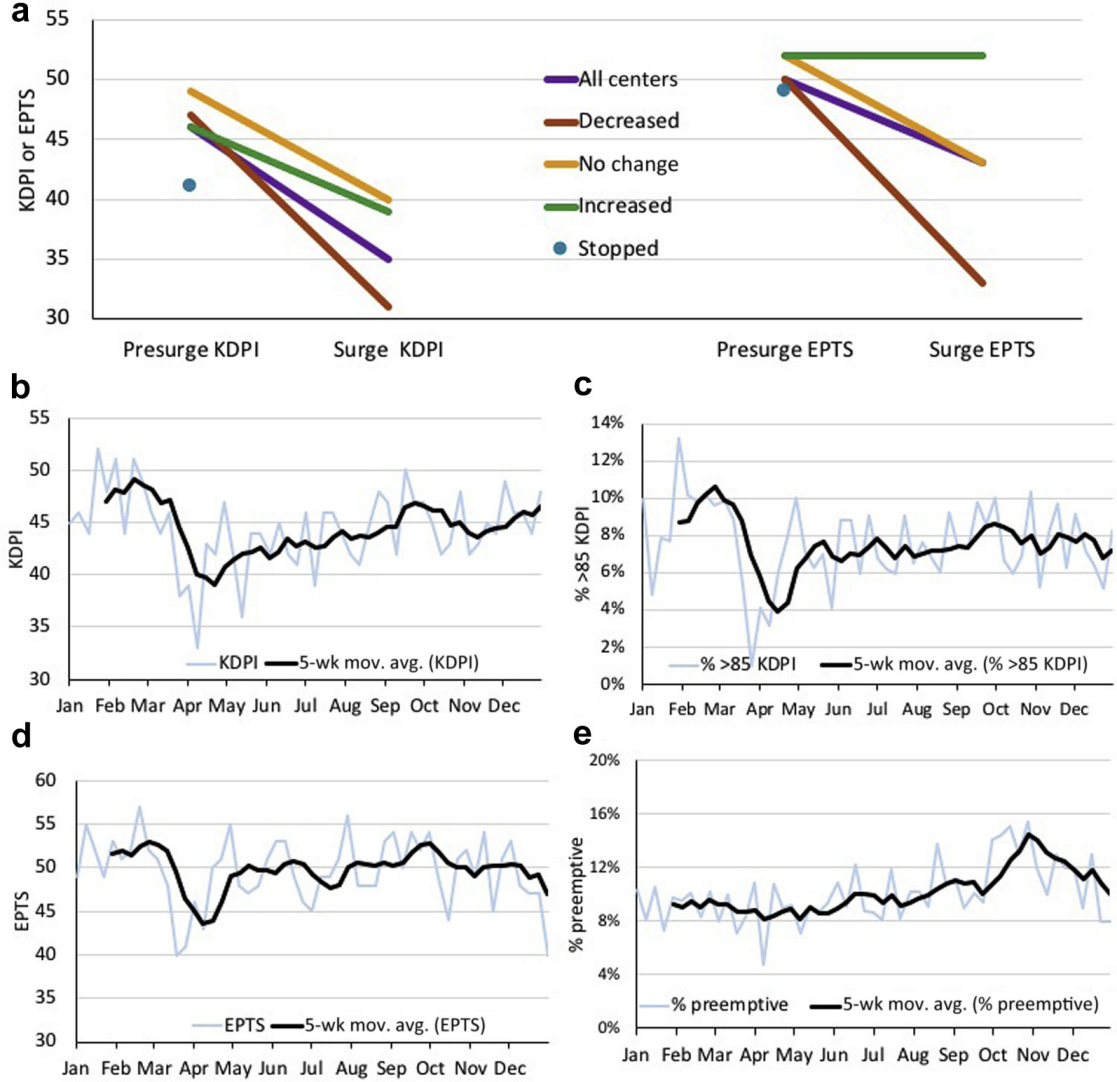
Change in KDPI and EPTS by transplant centers’ deceased donor kidney–only transplant volume during the presurge and initial surge (March 18–May 12, 2020) of the COVID-19 pandemic, trends of KDPI, % >85 KDPI, EPTS, and % pre-emptive recipients in 2020. EPTS, estimated post-transplant survival scores; KDPI, kidney donor profile index; 5-wk mov. avg., 5-week moving average.

During the initial COVID-19 surge (March 18–May 12, 2020) in the United States, most transplant centers either stopped (*n* = 43, 20%) or reduced (*n* = 91, 42%) the number of deceased donor kidney transplants being performed. Although some transplant centers (*n* = 38, 18%) maintained relatively stable transplant activity (within ±25% of their presurge transplant volume), the remaining centers (*n* = 44, 20%), at least one in each of the OPTN regions, increased their deceased donor transplant activity during the surge (Supplementary Figure S5). However, the median kidney donor profile index (KDPI) dropped up to 14 percentage points during the surge compared to presurge (*P* < 0.0001), from a median of 47 (interquartile range: 26–69) to a low of 33 (interquartile range: 18–55) in the week beginning April 8, 2020. Centers that decreased or had no change in their transplant volume had significantly lower KDPI during the surge (*P* < 0.0001 and *P* < 0.05, respectively), whereas centers that increased their transplant volume did not have a significant decrease in the KDPI of organs transplanted (*P* = 0.97; Figure 1a). Overall, after the initial surge there was an increase in KDPI, but it remained below the prepandemic median in the final quarter of 2020 (*P* < 0.001; Figure 1b). Utilization of KDPI >85% kidneys also decreased during the initial surge period (*P* < 0.0001) followed by a partial recovery afterward but remains significantly below prepandemic levels (*P* < 0.01; Figure 1c).

### Transplant Center Patient Selection

Along with the increased selectivity of kidneys transplanted during the initial surge, centers also transplanted patients with better estimated post-transplant survival scores (EPTS). The median EPTS prior to the surge was 52 (interquartile range: 23–79), and it dropped to 47 (interquartile range: 19–76) during the surge (*P* < 0.001) but returned to presurge levels by the end of 2020 (Figure 1d). Although this overall reduction in EPTS was largely driven by the centers that decreased their transplant activities, as the centers that maintained or increased their deceased donor kidney transplant volume during the surge did not have significantly different recipient EPTS compared with the presurge period (*P* = 0.06 and *P* = 0.36, respectively; Figure 1a). Notably, although the relative proportion of pre-emptive recipients did not change at the initial surge (*P* = 0.34), there was a significant and sustained increase in the proportion and absolute number of pre-emptive deceased donor transplants until the last quarter of 2020 compared with pre-surge (*P* < 0.001; Figure 1e). The nonlocal utilization of deceased donor kidney was not significantly different during the surge (31%, *P* = 0.31) but was significantly lower in the final quarter of 2020 (27%, *P* < 0.05) compared with presurge patterns (29%).

## Discussion

Our data highlight the pandemic’s effects on deceased donor kidney utilization and transplantation practices in the United States in 2020. Although transplant activity decreased dramatically during the spring surge in the United States, some centers maintained or increased their deceased donor transplant activity, with at least 1 transplant center in each OPTN region increasing its transplant volume. The decrease in the KDPI of transplanted kidneys and the EPTS of recipients is evidence of increased selectivity of both organs and recipients during the pandemic, especially for centers that decreased their transplant activities, underscoring the potentially subjective nature of some allocation decisions and the ability of centers to make informed choices. Although this abrupt change of transplant center behaviors suggests wide recognition of the need for caution early in the pandemic, it is notable that the median KDPI has not yet returned to prepandemic levels and pre-emptive transplants are still trending up as of the end of the year.

Although the discard rate was high throughout the surge period, the discard rate was lower post surge, and the annual discard rate was 21%, suggesting that improved use rates later in the year balanced out the higher numbers of weekly discards earlier in the year. The donor characteristics of the discarded and nonprocured kidneys remained consistent with previous years, but the most common reason noted for discard changed from “biopsy findings” to “no recipient located” in 2020.^5^^,^^6^ The sharp increase of “no recipient located” as the reason for discard from an average of 47% in January and February 2020 to up to 60% during the initial surge underscored the challenges of successful organ placement in the midst of a pandemic.^5^^,^^6^ Discard rates and procurement rates varied considerably across the country but were most prominently observed in region 9 (New York, western Vermont), consistent with geographic patterns of COVID-19 spread early in the pandemic. Our findings underscore the ability of centers to adjust their thresholds for which patients and organs they would consider for transplantation within the framework of the current allocation system by turning down deceased donor organ offers. The marked increase in organ discards potentially represents hundreds of missed opportunities for transplantation and emphasizes the need to develop contingency plans that would allow the continued use of deceased donor organs in circumstances such as future pandemics.

## Supplementary Material

**Supplementary Methods**

**Table S1.** Categorized reasons for discard of 5010 deceased donor kidneys in 2020.

**Table S2.** Deceased donor characteristics by organ dispositions during presurge and postsurge periods.

**Figure S1.** Counts of deceased donors and proportion of kidneys not procured or discarded by week (dotted line) in 2020 and with a 5-week moving average (solid line).

**Figure S2.** Counts of deceased donors and proportion of recovered kidneys that were discarded by week as a 5-week moving average in 2019 (green) and 2020 (orange and blue).

**Figure S3.** Counts of deceased donors and proportion of recovered kidneys that were discarded by week as a 5-week moving average with counts of weekly new COVID-19 cases.

**Figure S4.** Reported reasons for discarding deceased donor kidneys by month of 2020.

**Figure S5.** Change in transplant centers’ deceased donor kidney-only transplant volume during the initial surge of the COVID-19 pandemic.
