# Supplementary material for: Deceased Donor Kidneys Utilization and Discard Rates During COVID-19 Pandemic in the United States
Source: Kidney Int Rep. 2021 Jun 19;6(9):2463–7. doi: 10.1016/j.ekir.2021.06.002 (PMC8419126; doi:10.1016/j.ekir.2021.06.002)
Supplement: Supplementary File (PDF) [file mmc1.pdf]

Supplementary Material

Supplementary Methods

This study used national registry data from the UNOS Standard Transplant Analysis and Research files based on the OPTN database as of March 5, 2021. The transplant study cohort included all deceased donor kidney-only transplants in 2020 and the donor cohort included all deceased donors with a donation date in 2020. We calculated the sum of deceased donors, the sum of kidneys recovered for transplantation, and the sum of kidneys transplanted per week of 2020 beginning with January 1st, 2020 as the start of week 1. We plotted time trends of the weekly number of deceased donors, the proportion of kidneys not procured, and the proportion of recovered kidneys that were discarded (Supplementary Figure 1) with a 5-week moving average. We also plotted the number of deceased donors and the proportion of recovered kidneys that were discarded in 2019 for comparison with 2020 (Supplementary Figure 2) using 5-week moving averages. For comparison of donor and discard trends over time in 2020 against COVID-19 case counts, we used CDC state-level COVID-19 case surveillance data.[1] We aggregated the number of new cases per week in 2020 by OPTN region. Since Vermont is divided between region 1 and region 9, for simplicity all cases in Vermont were assigned to region 1.

For analysis we defined an "initial surge" period of COVID-19 in the United States as weeks 12-19, corresponding to March 18, 2020 through May 12, 2020. We calculated the total number of deceased donor transplants performed per week of 2020 and the sum of deceased donor transplants by transplant center in the pre-surge period (weeks 1-11) compared to the initial surge period (weeks 12-19). We classified centers into 4 groups based on their transplant volume before and during the initial surge: (1) centers that stopped transplanting entirely, with 0 deceased donor transplants performed during the surge, (2) centers that maintained about the same transplant volume during the surge, defined as within ±25% of their pre-surge transplant volume, (3) centers that decreased transplant volume by >25%, and (4) centers that increased transplant volume by >25%.

To examine how organ utilization and recipient selection behaviors may have changed throughout 2020, we calculated and plotted the median (interquartile range) kidney donor profile index (KDPI) and estimated post-transplant survival score (EPTS) of deceased donor transplants each week of 2020, along with the proportion of transplants from a donor with KDPI>85%, the proportion of transplants from a non-local donor, and the proportion of transplants for a preemptive recipient, defined as the absence of a reported dialysis start date and the absence of "Yes" for the pre-transplant dialysis variable and the regularly administered dialysis waitlist variable. We compared EPTS and KDPI pre-surge (weeks 1-11) versus during the initial surge (weeks 12-19) to determine whether they changed during the surge, and tested pre-surge versus the fourth quarter of 2020 to determine whether utilization patterns had returned to pre-surge levels. We used the chi-squared test to compare the proportion of KDPI>85% kidneys, non-local transplants, and preemptive recipients pre-surge versus surge and pre-surge versus quarter 4 of 2020. We also used the sign test for matched pairs to compared the center-level median KDPI and EPTS pre-surge versus initial surge stratified by volume change group.

Finally, we examined the disposition (transplant, discard, or non-procurement) of deceased donor kidneys in all of 2020 and during the initial surge period by donor characteristics, excluding kidneys without consent and kidneys recovered for research purposes. Of note, when examining overall organ disposition the proportion of kidneys discarded among all consented donor kidneys is slightly different from discard rates described above, which are calculated only among the kidneys procured for

transplantation. We also examined the disposition of deceased donor kidneys in the pre-surge period and in the post-surge period (Supplementary Table 2). Donor characteristics included age, gender, race/ethnicity (as reported in the UNOS database as a single variable, where we grouped Asian, American Indian/Alaska native, Native Hawaiian/other Pacific Islander, and Multiracial into Other), BMI, donation after circulatory death (DCD), absence of a negative Hepatitis C nucleic acid amplification test (HCV NAT) or anti-CMV test result, terminal creatinine, proteinuria, history of diabetes, history of hypertension, cigarette use (past or present), public health service increased risk designation, Kidney Donor Risk Index (KDRI) rao, KDPI, whether a biopsy was performed, and OPTN region. We compared whether organ disposition differed by level of categorical variables using the chi-squared test or whether there was a difference in median of continuous variables across disposition groups within each time period using the Kruskal-Wallis test. We categorized the reasons for discarding kidneys according to Supplementary Table 1 and examined the distribution of discard categories by month in 2020. Analyses were conducted using Stata MP 15.1 (Stata Corp, College Station, TX) and two-sided alpha of 0.05 determined statistical significance.

Supplementary Reference

1. Centers for Disease Control and Prevention, COVID-19 Response. United States COVID-19 Cases and Deaths by State over Time. Data last updated May 14, 2021. Data downloaded May 15, 2021. URL: https://data.cdc.gov/Case-Surveillance/United-States-COVID-19-Cases-and-Deaths-by-State-o/9mfq-cb36

Supplementary Table 1. Categorized reasons for discard of 5,010 deceased donor kidneys in 2020

Category	Discard Code	Description	N	%
Extended ischemia	601	Too old on pump	12	0.24
	602	Too old on ice	34	0.67
	611	Warm ischemic time too long	23	0.46
No recipient located	620	No recipient located - list exhausted	2,610	51.67
Biopsy findings	614	Biopsy findings	1,026	20.31
Donor history	606	Donor Medical history	39	0.77
	607	Donor social history	2	0.04
	610	Positive Hepatitis	8	0.16
	617	Infection	5	0.10
	618	Diseased organ	92	1.82
Organ damage	603	Vascular damage	48	0.95
	604	Ureteral damage	11	0.22
	612	Organ trauma	50	0.99
Anatomical abnormality	613	Organ not as described	17	0.34
	619	Anatomical abnormalities	186	3.68
Poor function	616	Poor organ function	247	4.89
	605	Inadequate urine output	4	0.08
Other/missing	615	Recipient determined to be unsuitable for transplant in operating room	18	0.36
	699	Other, specify	616	12.20
	missing	Missing	3	0.06

Supplementary Figure 1. Counts of deceased donors and proportion of kidneys not procured or discarded by week (dotted line) in 2020 and with a 5-weeks period moving average (solid line)

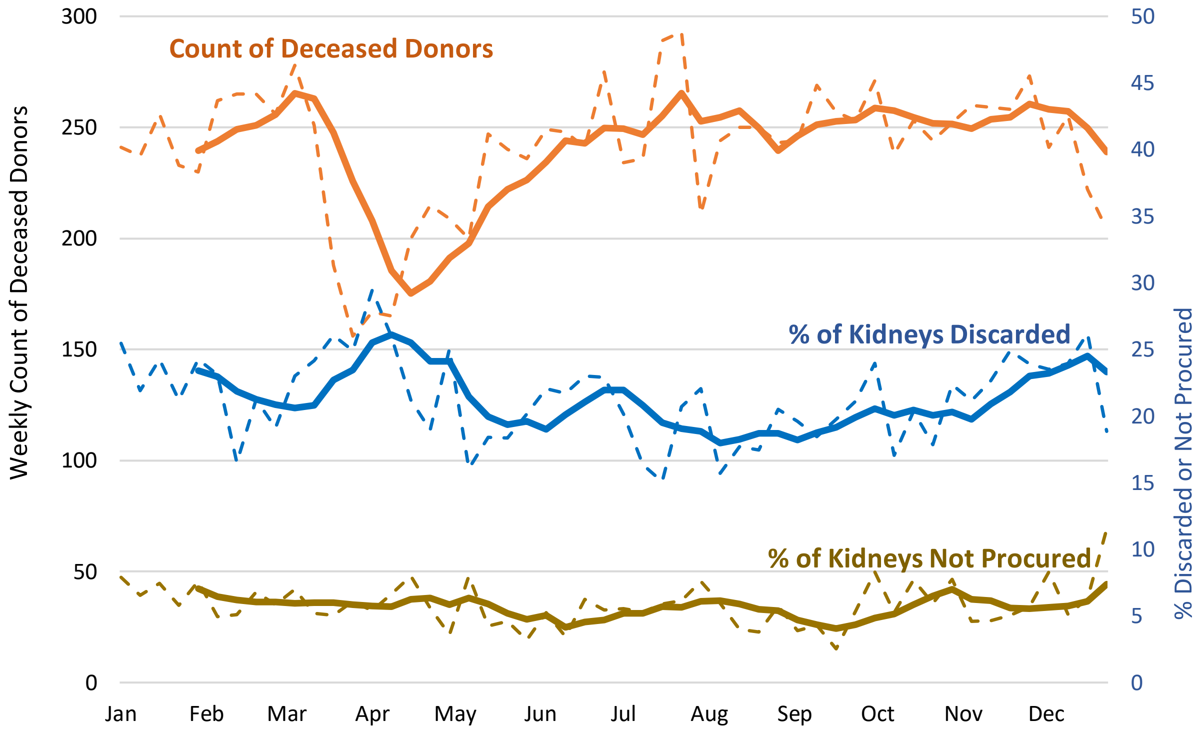

Supplementary Figure 2. Counts of deceased donors and proportion of recovered kidneys that were discarded by week as a 5-weeks period moving average in 2019 (green) and 2020 (orange and blue)

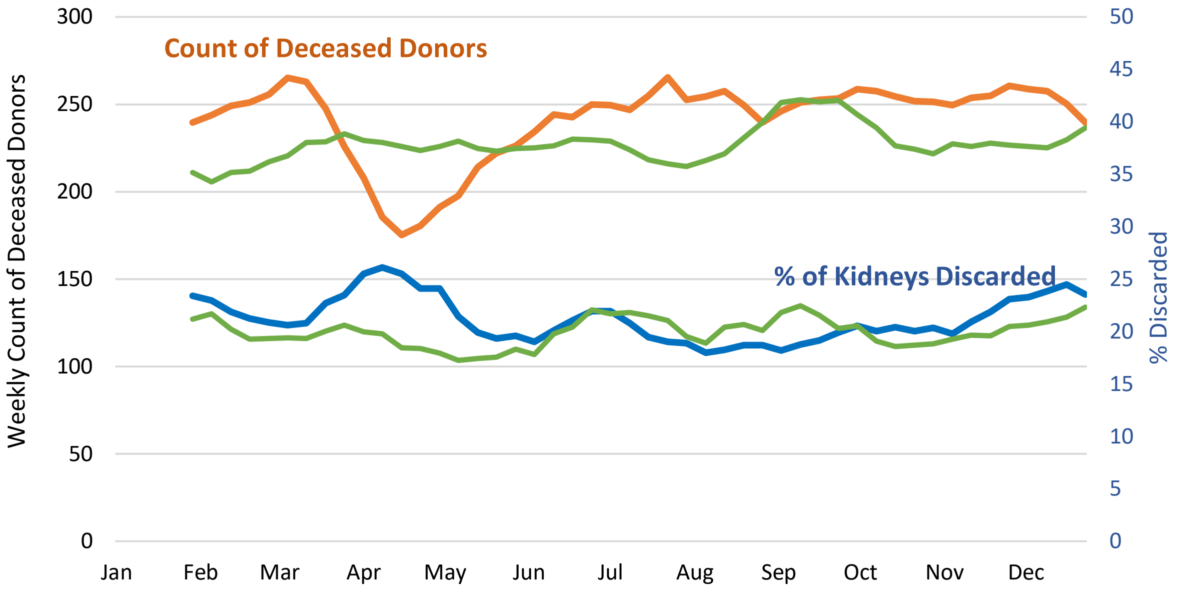

Supplementary Figure 3. Counts of deceased donors and proportion of recovered kidneys that were discarded by week as a 5-weeks period moving average with counts of weekly new COVID-19 cases

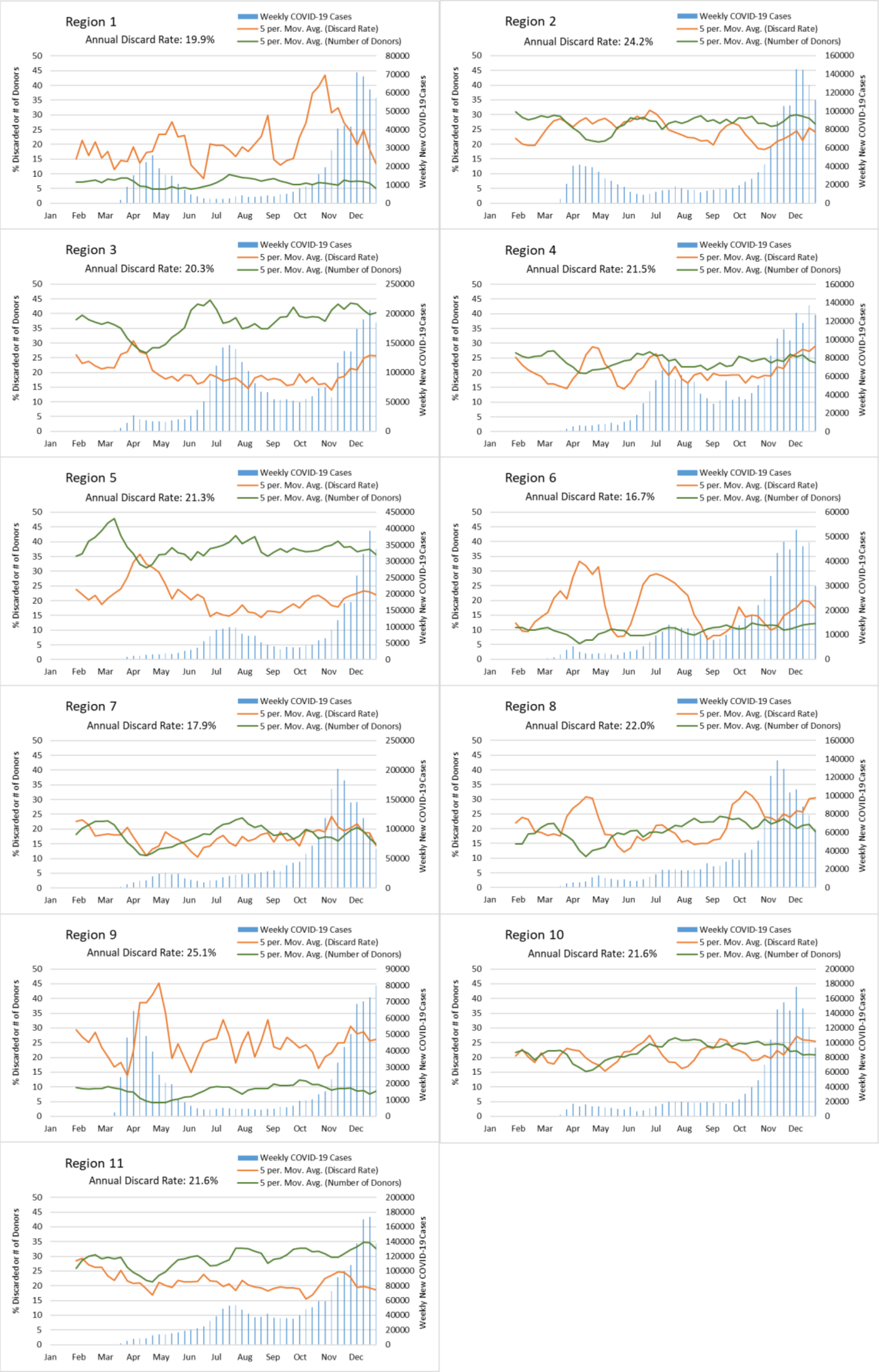

Supplementary Figure 4. Reported reasons for discarding deceased donor kidneys by month of 2020

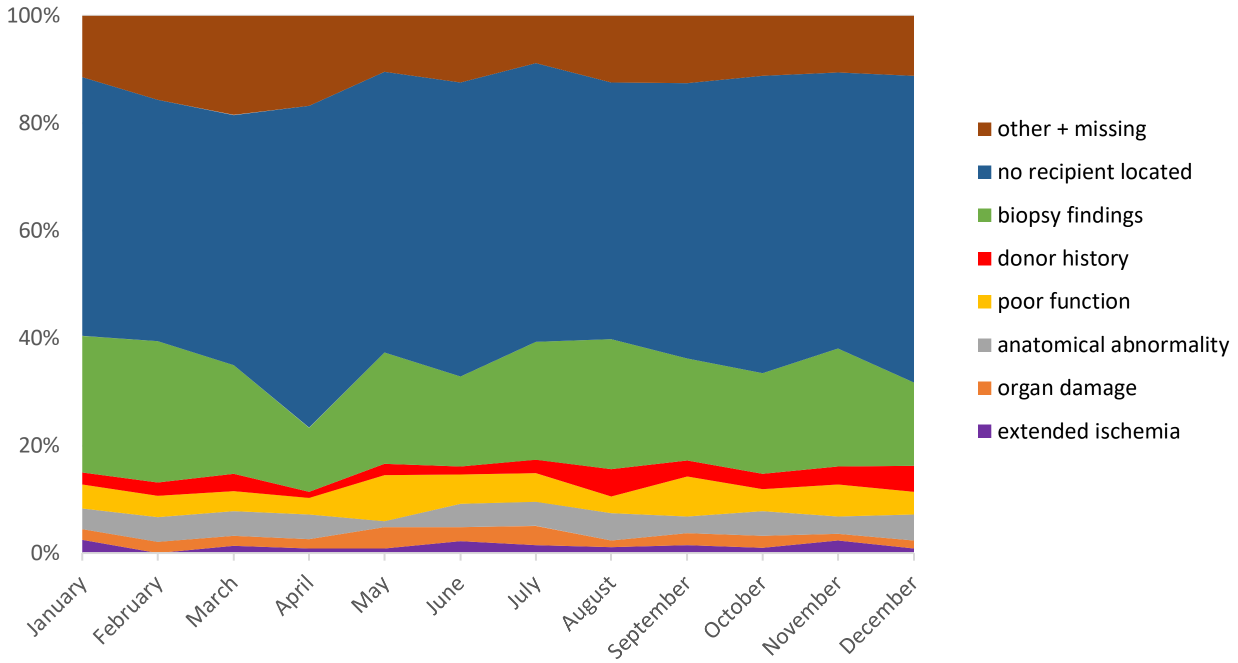

Supplementary Figure 5. Change in transplant centers' deceased donor kidney-only transplant volume during the initial surge of the COVID-19 pandemic

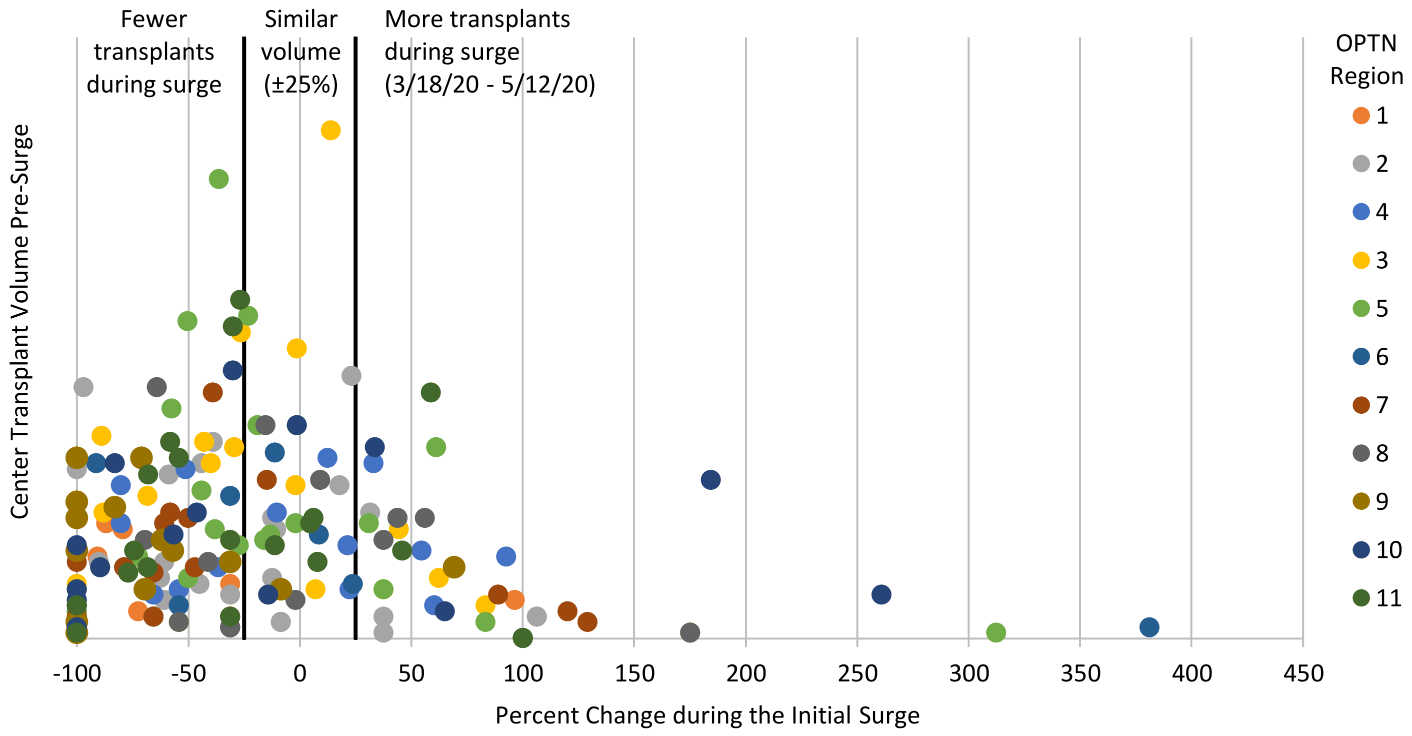

Supplementary Table 2: Deceased donor characteristics by organ dispositions during pre-surge and post-surge periods

Donor characteristics<br>Median (IQR) or Row %	Pre-Surge: Weeks 1-11<br>Transplanted<br>(n = 4,045)	 Discarded<br>(n = 1,150)	 Not Procured<br>(n = 289)	Post-Surge: Weeks 20-53<br>Transplanted<br>(n = 12,476)	 Discarded<br>(n = 3,247)	 Not Procured<br>(n = 793)
	73.8%	21.0%	5.3%	75.5%	19.7%	4.8%
Age	39 (28 - 51)	56 (48 - 63)	56 (45 - 66)	37 (27 - 50)	56 (45 - 62)	53 (43 - 64)
Gender						
Female	1,497 (71%)	504 (24%)	115 (5%)	4,522 (72%)	1,423 (23%)	308 (5%)
Male	2,548 (76%)	646 (19%)	174 (5%)	7,954 (78%)	1,824 (18%)	485 (5%)
Race/ethnicity						
White	2,668 (74%)	772 (21%)	162 (4%)	8,207 (76%)	2,278 (21%)	358 (3%)
Black	524 (66%)	197 (25%)	75 (9%)	1,878 (71%)	498 (19%)	267 (10%)
Hispanic	686 (80%)	132 (15%)	40 (5%)	1,885 (79%)	345 (15%)	148 (6%)
Other	167 (73%)	49 (21%)	12 (5%)	506 (78%)	126 (19%)	20 (3%)
BMI	27.4 (23.5 - 32.3)	29.4 (24.7 - 34.2)	28.0 (25.2 - 32.6)	27.1 (23.3 - 31.9)	28.7 (24.4 - 34.4)	28.6 (24.8 - 34.8)
DCD	1,096 (74%)	354 (24%)	28 (2%)	3,242 (75%)	1,043 (24%)	49 (1%)
HCV NAT+	273 (74%)	82 (22%)	15 (4%)	778 (74%)	241 (23%)	31 (3%)
CMV+	2,433 (72%)	742 (22%)	195 (6%)	7,961 (75%)	2,075 (20%)	534 (5%)
COVID-19 NAT or other test positive	0 (0%)	0 (0%)	0 (0%)	13 (46%)	15 (54%)	0 (0%)
COVID-19 antibody test positive	0 (0%)	0 (0%)	0 (0%)	18 (75%)	6 (25%)	0 (0%)
Terminal creatinine	1.0 (0.7 - 1.4)	1.6 (1.0 - 3.0)	3.8 (1.7 - 6.6)	0.9 (0.7 - 1.4)	1.5 (0.9 - 2.7)	4.3 (1.8 - 6.8)
Proteinuria	1,958 (68%)	690 (24%)	214 (7%)	6,731 (71%)	2,109 (22%)	578 (6%)
History of hypertension	1,167 (56%)	728 (35%)	205 (10%)	3,292 (56%)	2,009 (34%)	590 (10%)
History of diabetes	356 (45%)	312 (39%)	125 (16%)	967 (45%)	849 (40%)	323 (15%)
Cigarette use (past or present)	816 (65%)	368 (30%)	63 (5%)	2,377 (65%)	1,110 (30%)	190 (5%)
History of IV drug use	620 (83%)	101 (14%)	27 (4%)	1,727 (82%)	325 (16%)	43 (2%)
Public Health Services-Increased Risk (PHS-IR)	1,133 (76%)	236 (16%)	119 (8%)	3,511 (77%)	706 (16%)	320 (7%)
KDRI Rao	1.2 (1.0 - 1.5)	1.9 (1.5 - 2.2)	2.1 (1.6 - 2.4)	1.2 (1.0 - 1.5)	1.8 (1.5 - 2.2)	2.0 (1.6 - 2.5)
KDPI	45 (23 - 68)	86 (68 - 94)	91 (74 - 97)	42 (21 - 65)	83 (65 - 93)	89 (69 - 97)
Biopsy performed						
Yes	1,893 (68%)	1,011 (32%)	0 (0%)	6,256 (68%)	2,902 (32%)	3 (0%)
No	1,893 (85%)	139 (6%)	193 (9%)	6,220 (87%)	344 (5%)	598 (8%)
OPTN region						
1	132 (79%)	27 (16%)	9 (5%)	365 (79%)	93 (20%)	6 (1%)
2	484 (73%)	150 (23%)	27 (4%)	1,323 (72%)	419 (23%)	100 (5%)
3	572 (71%)	175 (22%)	62 (8%)	1,969 (75%)	459 (18%)	188 (7%)
4	438 (76%)	108 (19%)	31 (5%)	1,200 (75%)	335 (21%)	62 (4%)
5	653 (73%)	204 (23%)	41 (5%)	1,937 (78%)	455 (18%)	88 (4%)
6	184 (81%)	31 (14%)	11 (5%)	566 (82%)	114 (16%)	12 (2%)
7	334 (76%)	88 (20%)	18 (4%)	947 (78%)	194 (16%)	75 (6%)
8	286 (73%)	75 (19%)	33 (8%)	1,026 (74%)	305 (22%)	61 (4%)
9	146 (70%)	43 (21%)	19 (9%)	444 (71%)	145 (23%)	35 (6%)
10	373 (77%)	96 (20%)	16 (3%)	1,159 (74%)	335 (22%)	63 (4%)
11	443 (72%)	153 (25%)	22 (4%)	1,540 (76%)	393 (19%)	103 (5%)

+ HCV NAT and CMV are counted if the test results were positive, indeterminate, pending, or not done

Missing values excluded: BMI (50), proteinuria (220), hypertension (304), diabetes (304), cigarette use (592), IV drug use (396), KDRI/KDPI (2), biopsy (289

Abbreviations: IQR, interquartile range; BMI, body mass index; DCD, donation after circulatory death; HCV, hepatitis C virus; NAT, nucleic acid amplificatior
